# ‘ *BJUI* Clinical Dilemma’: the overactive bladder

**DOI:** 10.1111/bju.70043

**Published:** 2025-10-21

**Authors:** Oliver Gross, Marcio A. Averbeck, Stefania Musco, Véronique Phé, Desiree Vrijens, Blayne Welk, Glenn T. Werneburg, Thomas M. Kessler

**Affiliations:** ^1^ Department of Neuro‐Urology, Balgrist University Hospital University of Zürich Zürich Switzerland; ^2^ Department of Urology Moinhos de Vento Hospital Porto Alegre Brazil; ^3^ Department of Neuro‐Urology Azienda Ospedaliera Universitaria Careggi Florence Italy; ^4^ Department of Urology, Assistance Publique‐Hôpitaux de Paris, Tenon Academic Hospital Sorbonne University Paris France; ^5^ Department of Urology Maastricht University Medical Center Maastricht The Netherlands; ^6^ Department of Surgery and Epidemiology and Biostatistics Western University London Ontario Canada; ^7^ Department of Urology University of Michigan Ann Arbor MI USA; ^8^ Department of Urology Cleveland Clinic Foundation Cleveland OH USA

**Keywords:** overactive bladder, OAB, individualised management, behavioural treatment, pharmacological treatment, neuromodulation, botulinum toxin, surgery, shared decision‐making, clinical dilemma

**Abbreviations**
β_3_
: beta‐3
EAU: European Association of Urology
OAB: overactive bladder
SNM: sacral neuromodulation
(U)UI: (urgency) urinary incontinence

## Introduction

Overactive bladder (OAB) is a symptom‐defined clinical syndrome characterised by urinary urgency, usually accompanied by increased daytime frequency and nocturia, with or without urgency urinary incontinence (UUI), in the absence of UTI or other identifiable pathology [1, 2, 3, 4]. In adults aged >40 years, OAB affects 9–43% of women and 7–27% of men, with women consistently showing higher prevalence [5, 6]. A survey of adults conducted in five countries demonstrated an overall prevalence of OAB symptoms of 12% [7, 8]. OAB substantially reduces quality of life, increases the risk of depression and falls, and imposes a significant economic burden on healthcare systems [7, 9]. Clinical management of OAB remains challenging. Conventional diagnostic evaluation often fails to correlate symptoms with objective findings: up to 50% of patients with urgency symptoms do not have detrusor overactivity on urodynamic investigation, and vice versa [1, 10]. Conservative therapies such as behavioural therapy, pelvic floor physiotherapy and medication are frequently ineffective or poorly tolerated due to side effects and have limited long‐term adherence [11, 12]. Patients with persistent symptoms often face complex decisions involving minimally invasive treatment modalities such as intradetrusor botulinum toxin injections or sacral neuromodulation (SNM), despite substantial diagnostic uncertainty regarding the underlying pathophysiology. This ‘*BJUI* Clinical Dilemma’ aims to explore a representative case of refractory OAB and the diagnostic and therapeutic uncertainties that surround it. A structured case vignette will serve as the basis for expert commentary on a range of diagnostic and management options. This approach highlights the challenges of personalised decision‐making in a field where empirical practice often fills gaps in mechanistic understanding, and emphasises the importance of shared, patient‐centred strategies in managing this common but complex condition.

## CASE Vignette

**Fig. 1:**
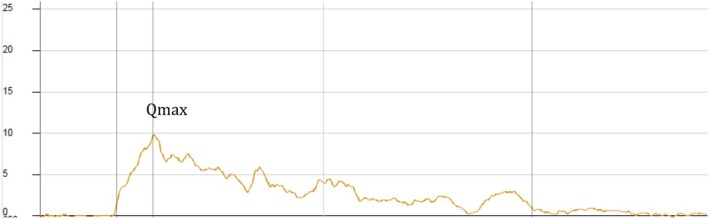
Free uroflowmetry with a flattened and elongated curve and post‐void residual of 65 mL. Voided volume = 265 mL, maximum urinary flow rate (Q_max_) = 10 mL/s.

**Fig. 2:**
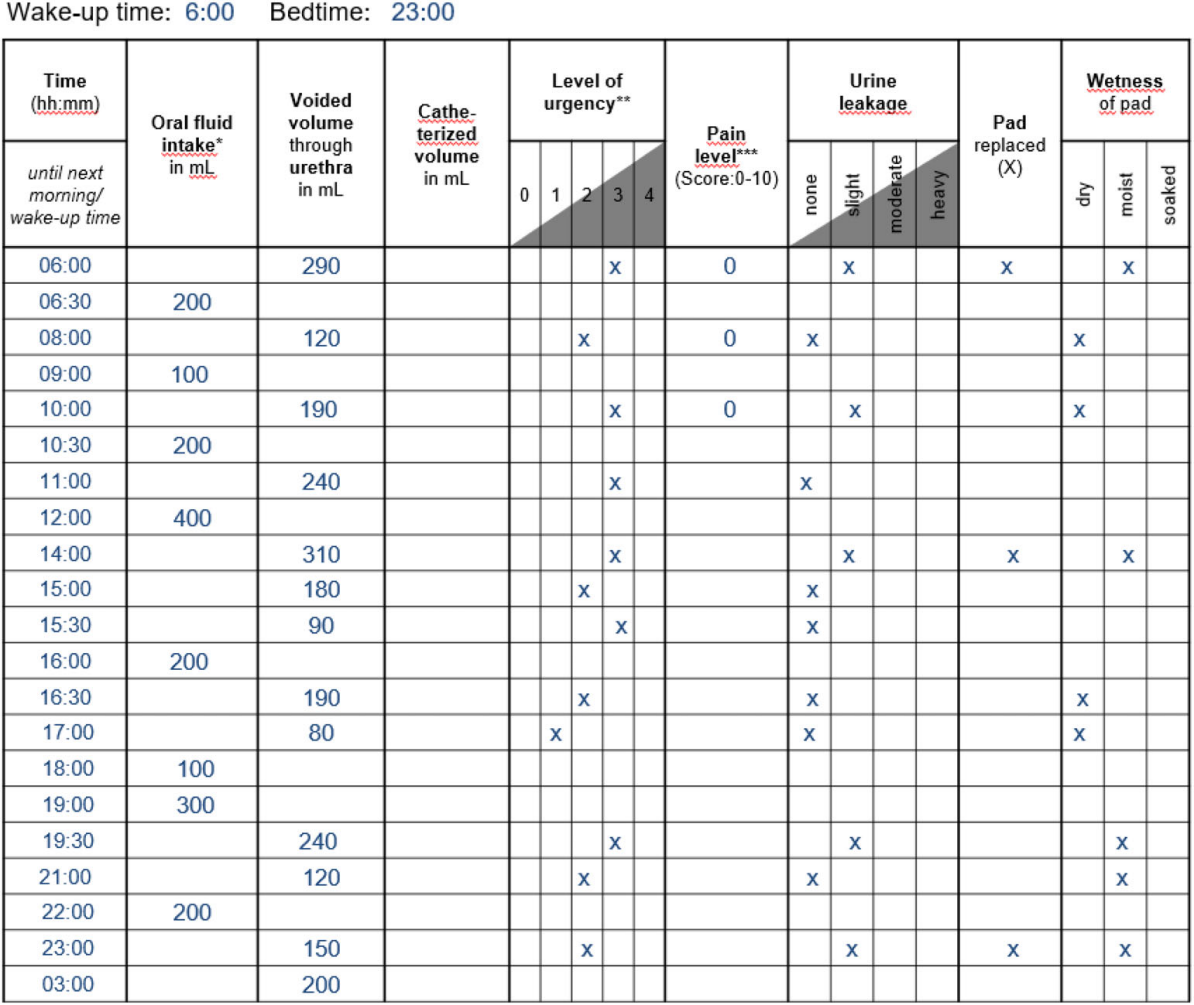
Representative day from the patient's bladder diary, which was kept over a period of 3 days.

**Fig. 3:**
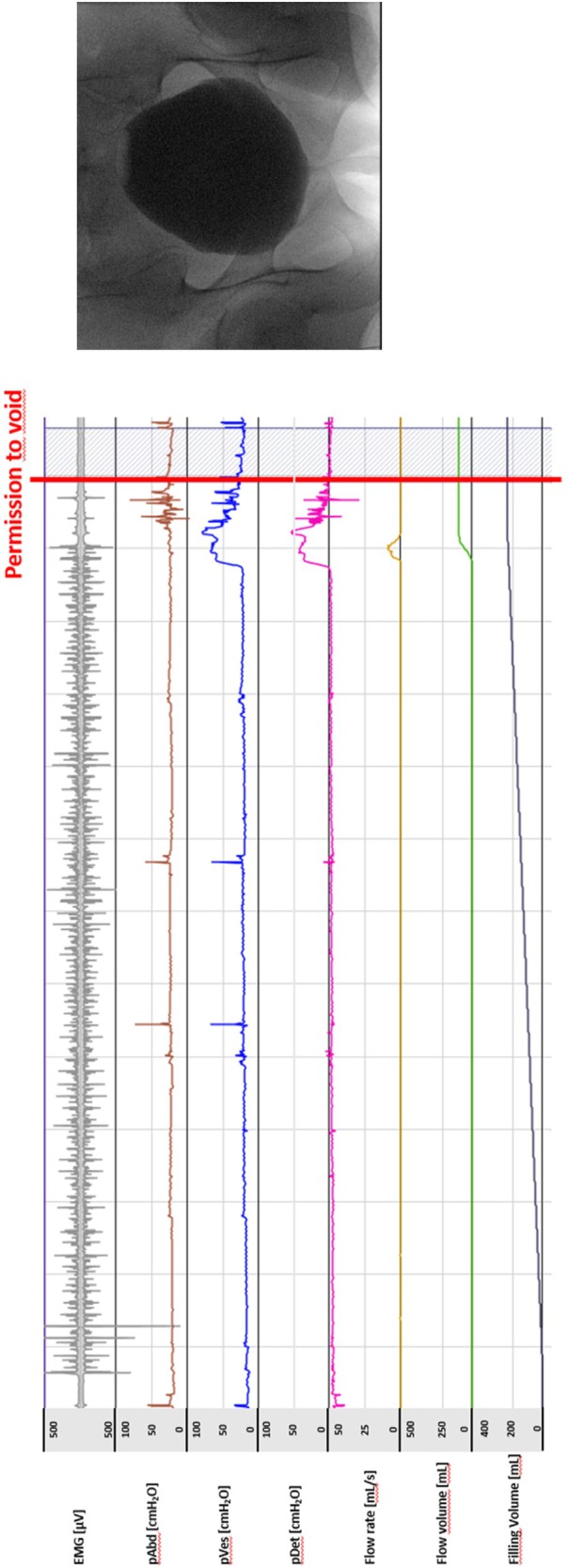
Video‐urodynamic findings of the patient with detrusor overactivity followed by detrusor overactivity UI. EMG, electromyography; pAbd, abdominal pressure; pDet, detrusor pressure; pVes, vesical pressure.

A 36‐year‐old woman presents with a 15‐month history of bothersome urinary urgency and UUI. She reports voiding 12 times/day, with no more than one nocturnal episode. She describes a pronounced urgency sensation that is occasionally accompanied by UUI, particularly in stressful or unfamiliar environments. She denies dysuria, haematuria, or pelvic pain. Her medical history is unremarkable. She had no prior urological surgeries and no history of recurrent UTIs. She is nulliparous, takes no medications, and denies smoking or excessive caffeine intake. Physical examination, including focused neurological assessment and pelvic floor inspection, is unremarkable. Free uroflowmetry demonstrated a maximum urinary flow rate of 10 mL/s with a total voided volume of 265 mL. Post‐void residual was 65 mL (Fig. 1). A 3‐day bladder diary revealed an average of 12 voids/24 h and one nightly void, with a mean (range) voided volume of 195 (80–310) mL. All voiding episodes were accompanied by a moderate to strong sensation of urgency. Total fluid intake averaged 1700 mL/24 h (Fig. 2). Urine analysis showed no evidence of infection. Cystoscopy showed normal urethral and bladder anatomy without signs of inflammation, tumour, or bladder trabeculation. Video‐urodynamics demonstrated detrusor overactivity UI and the bladder capacity was within the normal range. Maximum detrusor pressure amplitudes during the storage phase reached 52 cmH_2_O. The pressure–flow study showed no evidence of obstruction (Fig. 3).

## INITIAL Management Options

### Behavioural Interventions

Behavioural therapy is a form of conservative management (non‐pharmacological and non‐surgical) with favourable cost and safety profiles and is supported by guideline consensus [3, 4, 8]. The core behavioural strategies for OAB include bladder training with timed voiding, fluid modification, urgency suppression techniques, and pelvic floor muscle training. While evidence strength varies across these modalities, they remain widely used given their low risk and accessibility.

Bladder training is an educational programme for patients to re‐establish voiding habit control through a regimen of progressively increased intervals between voids. A Cochrane review concluded that bladder training may cure or improve OAB relative to no therapy, and that it may be more effective and have fewer side effects than antimuscarinic agents [13]. Fluid management aims to reduce known bladder irritants and optimise fluid volume intake. A systematic review found positive associations between fluid intake and caffeine use with urinary frequency and urgency [14], but further evidence is needed to definitively demonstrate a causal link or to measure symptomatic improvement following fluid optimisation. Urgency suppression techniques have been described and include a series of ‘quick flick’ pelvic floor muscle contractions, deep breathing, and/or mental distraction in the case of new onset urgency [15]. A supervised pelvic floor muscle training regimen aims to optimise voluntary contraction of the pelvic floor muscles, which in turn may inhibit detrusor contractions and reduce UI episodes [15]. While the evidence base for all of these techniques is low, given their very low risk and low cost, they should be offered early in the course of OAB therapy.

In this patient, a trial of behavioural therapy is very appropriate given her young age, absence of comorbidities, and recent symptom onset. A multimodal behavioural therapy regimen including bladder training, fluid modification, and guided pelvic floor muscle training should be offered. In terms of fluid modification, her 24‐h intake of 1700 mL appears appropriate, but the patient may benefit from assessment and elimination of potential bladder irritants such as caffeine [16]. Her night‐time symptoms are minimal, and thus reduction in late‐day fluid intake is unlikely to be necessary.

Similar to a trial of pharmacological therapy, patients should be encouraged to commit to such a structured programme for a minimum of 4–8 weeks, at which time symptoms may be reassessed [8]. Benefit may build gradually and is dependent on consistency. Implementation of such a regimen under the supervision of a continence nurse, pelvic health therapist, or advanced practice provider, may improve adherence and outcomes.

Despite the rationale for behavioural interventions, these are often limited in efficacy by factors including patient adherence and suboptimal instruction or guidance. In cases of incomplete response, these strategies should not be abandoned. Rather, they should be continued as adjunctive therapies alongside pharmacological or advanced interventions such as chemodenervation and neuromodulation.

### Beta‐3 (β_3_) Adrenergic Agonists

The β_3_ agonists are the most recently available oral medication class for the treatment of OAB. The β_3_ receptor in the bladder mediates detrusor relaxation, and the two clinically available medications (mirabegron and vibegron) are highly selective for the β_3_ receptor, with limited cross‐reactivity to β_1_ and β_2_ receptors [17].

The β_3_ agonists can be considered first‐line oral therapy for patients with bothersome OAB. This challenges the traditional paradigm in which antimuscarinics were often considered first‐line oral therapy due to their familiarity to physicians, their well‐established body of clinical evidence, and payer bias towards the lower costs of these medications compared to β_3_ agonists. The β_3_ agonists provide equally efficacious symptom management compared with antimuscarinics. This has been shown directly in a randomised trial where treatment‐naive people with OAB were given 4 week courses of tolterodine, mirabegron, or placebo in a random, blinded order, and were then asked to pick their preferred treatment; the result was an almost even split of participants choosing mirabegron and tolterodine, with the most commonly cited reason for selecting a specific medication being better perceived efficacy [18]. This is consistent with systematic reviews and network meta‐analyses, which generally show equal efficacy across all oral OAB medications [19].

While efficacy may be similar, β_3_ agonists have two distinct advantages compared to antimuscarinics. First, they have a considerably lower risk of causing dry mouth, which occurs threefold more frequently with antimuscarinics [20]. This may explain the increased medication persistence that is seen with β_3_ agonists compared to antimuscarinics [21]. Second, β_3_ agonists do not have cognitive concerns with their use. Studies have demonstrated an increased risk of dementia in people who use antimuscarinics compared to those that use β_3_ agonists [22]. It is important to note that initial concerns about possible serious cardiovascular side effects from β_3_ agonists, mediated by hypertension or cross‐reactivity with cardiac β receptors, have not born out in real‐world clinical studies [23].

The most recent AUA OAB guidelines no longer recommend delivering OAB therapies in a stepwise progression, and similarly the European Association of Urology (EAU) guidelines suggest using shared decision‐making when selecting a pharmacotherapy option [3, 4, 8]. Both guidelines provide strong endorsements for antimuscarinics and β_3_ agonists. Clinicians should use their clinical judgement, and the patient's preferences, when selecting an oral OAB medication. For the reasons outlined above, it makes sense in many cases to trial a β_3_ agonist as first‐line oral therapy.

### Antimuscarinics

In patients presenting with UUI and urodynamic detrusor overactivity, antimuscarinics represent a first‐line pharmacological treatment option. These agents act by antagonising muscarinic receptors in the detrusor, thereby reducing involuntary contractions during the storage phase and OAB symptoms [8]. In our clinical case of a 36‐year‐old woman with UUI, detrusor overactivity, and a modestly elevated post‐void residual, antimuscarinics may provide symptom relief while warranting close monitoring.

Antimuscarinics such as darifenacin, fesoterodine, imidafenacin, oxybutynin, propiverine, solifenacin, tolterodine, and trospium chloride have demonstrated efficacy in reducing urgency and UUI, improving quality of life and decreasing voiding frequency [12, 24]. Improved patient adherence to medical treatment has been reported in once‐daily and extended‐release dosing regimens [24, 25].

Common side effects of antimuscarinics include dry mouth, constipation, blurred vision, and dizziness, primarily due to non‐selective muscarinic blockade [26]. Of particular concern is the potential for cognitive impairment, especially with agents that cross the blood–brain barrier (such as oxybutynin). In contrast, trospium chloride, a quaternary amine, has limited CNS penetration, and may be preferred in patients at risk of cognitive decline – even though this risk is minimal in a healthy 36‐year‐old patient [27].

In the presence of a post‐void residual of 65 mL, the risk of urinary retention must be considered. Although this post‐void residual is below the threshold typically associated with complications, antimuscarinics may exacerbate voiding dysfunction by reducing detrusor contractility, potentially leading to increased post‐void residual or acute urinary retention [28]. Thus, medical follow‐up including repeat post‐void residual measurements are recommended after initiation of therapy.

### Early Combined Pharmacotherapy

This young patient presents with bothersome symptoms and therefore achievement of effective control with pharmacotherapy, after failed behavioural measurements, is very important in order to prevent the need for more invasive treatment.

There are several randomised, double‐blind phase II–III studies comparing combined therapy of solifenacin with mirabegron to monotherapy or placebo. These sponsored studies (SYMPHONY [ClinicalTrials.gov identifier: NCT01340027], SYNERGY [NCT01972841], and BESIDE [NCT01908829]) all show significant improvements for combined therapy compared to monotherapy and placebo [29, 30, 31]. The combined therapy was well tolerated and there was no significant difference in the reported side effects between monotherapy and combined therapy. The long‐term efficacy and safety is studied in the SYNERGY II (NCT02045862) study, a randomised, double‐blind controlled trial with a treatment time of 12 months [32]. Combined therapy of 5 mg solifenacin daily with 50 mg mirabegron daily led to less UI episodes and lower numbers of micturitions compared to monotherapy of either mirabegron or solifenacin and it was well tolerated. Long‐term safety of combination of antimuscarinics with mirabegron is confirmed by the MILAI II (NCT02294396) study, a phase IV study comparing mirabegron with the add‐on of either solifenacin 5 mg daily, propiverine 20 mg daily, imidafenacin 0.2 mg daily, or tolterodine 4 mg daily for 52 weeks [33]. The add‐on treatment with antimuscarinics was well tolerated and effective in improving the outcomes on bladder diaries and questionnaires.

Nevertheless, more recently, a non‐sponsored trial in patients with OAB revealed better treatment efficacy with the combined therapy of mirabegron with solifenacin compared to monotherapy, but a lower patient adherence due to side effects. We can argue that this is not applicable to our patient as the mean age in this study was 73 years [34].

The best option to reach a satisfactory treatment result with pharmacotherapy is with combined therapy and in this young woman with serious symptoms this should be considered early in the treatment process.

## CASE Vignette Continued

The patient reports that her symptoms have only marginally improved with conservative treatment. Behavioural strategies, including bladder training and fluid adjustment, were applied consistently over several weeks but did not lead to meaningful improvement of the symptoms. Pharmacotherapy with mirabegron was well tolerated but did not relevantly reduce urinary urgency and UUI. Two different antimuscarinics were each taken for only a few days and then discontinued due to severe dry mouth. Despite all these efforts, the patient continues to experience pronounced urgency and daytime frequency, occasionally accompanied by UUI. She remains highly motivated to pursue further treatment and is actively seeking advice on next steps.

## FURTHER Management Options

### Intradetrusor Botulinum Toxin Injections

Botulinum toxin A is widely recognised across international guidelines as a therapeutic option for refractory idiopathic UUI, with or without detrusor overactivity. Despite the availability of several formulations, United States Food and Drug Administration (FDA) approval has remained still restricted to onabotulinumtoxinA (Botox^®^; AbbVie Inc., North Chicago, IL, USA) since 2013 [8].

Clinical trials have demonstrated that, at 12 weeks, intradetrusor onabotulinumtoxinA injections (100 U Botox) achieve the greatest reductions in urgency, UUI and micturition frequency (≥50% reduction from baseline), as well as higher cure rates when compared with antimuscarinics or β_3_ agonists [35]. The mean duration of effect in idiopathic OAB is generally 6–9 months. However, repeated injections have shown sustained efficacy and safety in both medium‐ and long‐term follow‐up studies [36].

The standard injection technique involves the administration of 100 U Botox diluted in 20 mL sterile saline, distributed across 20 intradetrusor sites (1 mL/injection), under local anaesthesia or sedation using either rigid or flexible cystoscopy. The most frequent adverse events include UTI, urinary retention, and voiding dysfunction in addition to procedure‐related complications such as haematuria or pain [8, 35, 36]. To optimise efficacy and safety, alternative injection paradigms have been investigated. Trigone‐sparing injections may reduce the incidence of UTI, while lowering the number of injection sites without changing the total dose appears to decrease the risk of voiding dysfunction [37, 38]. Nevertheless, accurate estimates of UTI and urinary retention rates remain difficult due to heterogeneous study designs and the lack of standardised outcome definitions [39, 40]. Consequently, phenotyping strategies to identify patients who might benefit from antibiotic prophylaxis or other preventive measures remain insufficiently established [41, 42].

Treatment selection should be guided by a shared decision‐making process, weighing the relative benefits and risks of botulinum toxin A compared with SNM, as no therapeutic hierarchy is currently recommended [8, 36]. While detrusor overactivity does not appear to influence the efficacy of either therapy, in young women with dysfunctional voiding, the possibility of an underlying neurological pathology should be considered [43, 44]. In such cases, botulinum toxin A offers a reversible, minimally invasive initial option, providing an observational period during which neurological symptoms may become apparent and prompt further diagnostic assessment. By contrast, MRI‐conditional SNM systems are limited by specific MRI parameters, precluding the use of ultra‐high‐field imaging and potentially hindering early diagnosis of neurological disorders [45].

In women of childbearing age, reproductive plans should be addressed before initiating treatment. Patients should be counselled that no data currently confirm fetal safety in the context of SNM system implantation, and device deactivation throughout pregnancy is generally advised, with the potential for symptom exacerbation. Conversely, intradetrusor botulinum toxin injections may be performed before a planned conception, as available data do not suggest an increased risk of pregnancy complications or adverse fetal outcomes [46].

The main clinical concerns with intradetrusor botulinum toxin injections remain UTIs and urinary retention, even if in this case the patient can void spontaneously with a post‐void residual <100 mL [46]. Recurrent UTIs or the need for intermittent catheterisation represent leading causes of treatment discontinuation, which can substantially compromise the perception of improvement, satisfaction and adherence, particularly in those without prior experience of UTIs or catheter use [47].

### Neuromodulative Treatment

Neuromodulation plays an important role in the management of refractory OAB by modulating afferent and efferent neural control. Transcutaneous and percutaneous electrical nerve stimulation – including tibial nerve stimulation, offers a non‐invasive approach. Clinical studies have shown that both transcutaneous and percutaneous tibial nerve stimulation can reduce urgency, frequency, and UUI [48, 49]. SNM offers a permanent electrical stimulation of the sacral S3 (or S4) nerve root via an electrode connected to an implantable pulse generator. It is a well‐established treatment for refractory OAB [50, 51] and efficacy and safety of SNM have also been shown in selected patients with neurogenic lower urinary tract dysfunction [52]. In the present case, the combination of OAB and slightly elevated post‐void residual supports the rationale for SNM. Tibial nerve stimulation may serve as a non‐invasive option with minimal burden, while SNM offers the potential for long‐term efficacy.

## CASE Vignette Continued

After discussing further management options, the patient expresses interest in a treatment that could offer meaningful long‐term improvement without daily medication. Meanwhile, the patient also inquired about the option of vaginal laser therapy. However, as such an intervention should only be performed within a controlled study setting [3, 4], this option was not pursued due to the lack of eligibility for inclusion in a clinical trial. She is particularly intrigued by the possibility of SNM, given its reversibility and stepwise approach. However, she also asks about the efficacy and potential side effects of intradetrusor botulinum toxin injections. After a thorough counselling session, she agrees to undergo a test phase for SNM.

## SALVAGE Management Options

### Surgical Approaches

Surgery for idiopathic OAB is exceptional and should be reserved as a salvage option after failure of conservative, pharmacological, and minimally invasive therapies (including botulinum toxin and neuromodulation). Among operative strategies, augmentation cystoplasty can increase capacity and compliance, but high‐quality evidence in idiopathic OAB is limited and outcomes are consistently worse than in the neurological population. No randomised trial has compared augmentation with other treatments of idiopathic OAB. The EAU guidelines report long‐term continence and satisfaction around one‐half in mixed cohorts (neurological and non‐neurological population), with inferior results for idiopathic detrusor overactivity, and emphasise substantial complication burdens: need for intermittent catheterisation, recurrent UTIs, metabolic disturbances, stone formation, and rare but documented malignancy >10 years post‐augmentation, mandating life‐long follow‐up and patient ability/willingness to perform intermittent catheterisation [53]. Operative technique nuances (coronal vs sagittal bivalving) [54, 55] have not shown meaningful differences, and robot‐assisted augmentation appears to achieve functional results comparable to open surgery at the cost of longer operative time [56]. Detrusor myectomy (auto‐augmentation) demonstrates poor durability due to fibrosis and is not recommended [53]. Urinary diversion (e.g., ileal conduit or continent diversion) is a last resort for refractory OAB after multiple failed interventions or complex pelvic pathology; choice is individualised with thorough preoperative counselling and specialist stoma/continence nurse support [53].

## Discussion

Overactive bladder can present with a highly variable clinical course, sometimes lacking a direct correlation between symptoms and urodynamic findings [1, 10, 28]. This discordance presents a significant challenge for diagnosis and management, emphasising the need for an individualised, patient‐centred approach rather than a purely test‐driven strategy. The presented case exemplifies the diagnostic and therapeutic challenges in patients with refractory OAB and confirmed detrusor overactivity. Conservative strategies, such as behavioural therapy, remain the recommended starting point due to their favourable safety profile and accessibility [3, 4, 8, 13]. However, as in the present case, efficacy may be limited by patient adherence, symptom severity, and the absence of modifiable lifestyle factors. Pharmacological treatment with antimuscarinics or β_3_ agonists is supported by robust evidence for symptom reduction [8, 12, 24, 25, 26]. While antimuscarinics are effective, their anticholinergic side effects, particularly dry mouth and potential cognitive effects, may limit adherence [24, 26, 27]. The β_3_ agonists offer similar efficacy with better tolerability, but treatment response can remain insufficient [8, 57]. Combined therapy of antimuscarinics and β_3_ agonists has been shown to provide superior symptom control compared to monotherapy, with an acceptable safety profile [29, 30, 31, 32, 33]. Although our patient's intolerance to antimuscarinics precluded this option, early consideration of combined regimens may prevent progression to invasive interventions in selected cases [32]. Given their favourable safety profile and minimal invasiveness, non‐invasive neuromodulation techniques such as transcutaneous or percutaneous tibial nerve stimulation may represent a promising initial step in the neuromodulatory treatment algorithm [43, 44]. Botulinum toxin is highly effective in reducing urgency and UUI but carries a risk of transient urinary retention requiring intermittent catheterisation [28, 50]. SNM offers sustained symptom improvement and the advantage of a reversible test phase [50, 51, 52], making it particularly appealing to younger patients motivated to avoid long‐term medication. In the present case, the choice of SNM aligns with guideline recommendations and patient preference. Ultimately, the management of OAB requires a tailored, stepwise approach, integrating objective findings, symptom severity, patient values, and tolerability profiles. Early shared decision‐making is essential to balance treatment efficacy, invasiveness, and quality‐of‐life considerations. The case underscores the need for continued research into pathophysiological mechanisms to enable more precise, mechanism‐based therapies for OAB.

## Conclusions

- Overactive bladder is a heterogeneous condition requiring individualised diagnostic and therapeutic approaches.
- Urodynamics is essential to reveal the underlying dysfunction.
- An escalation from behavioural to pharmacological and eventually to minimally invasive therapies remains the cornerstone of OAB management.
- In refractory cases, both botulinum toxin and SNM are evidence‐based and effective options.
- Surgical interventions are rarely needed but may be considered as a salvage treatment option.
- Shared decision‐making and alignment with patient values and expectations are essential to successful management.

## Disclosure of Interests

The authors have no direct or indirect commercial financial incentive associated with publishing this article.
